# Multilevel analysis of the predictors of completion of the continuum of maternity care in Ethiopia; using the recent 2019 Ethiopia mini demographic and health survey

**DOI:** 10.1186/s12884-022-05016-z

**Published:** 2022-09-07

**Authors:** Gossa Fetene Abebe, Dereje Zeleke Belachew, Desalegn Girma, Alemseged Aydiko, Yilkal Negesse

**Affiliations:** 1 Department of Midwifery, College of Medicine and Health Sciences, Mizan-Tepi University, P.O.Box-260, Mizan Teferi, Ethiopia; 2 Department of Public health, College of Medicine and Health Sciences, Debre Markos University, Debre Markos, Ethiopia

**Keywords:** Continuum of care, Maternity services, Predictors, Multilevel analysis, Ethiopia

## Abstract

**Background:**

Despite the significant benefit of the continuum of care to avert maternal and neonatal mortality and morbidity, still the dropout from the continuum of care remains high and continued to become a challenge in Ethiopia. Therefore, this study aimed to assess the level of completion along the continuum of maternity care and its predictors among reproductive-age women in Ethiopia.

**Methods:**

A secondary data analysis was done using the 2019 mini Ethiopian demographic health survey. A total weighted sample of 2,905 women aged 15–49 years who gave birth in the last five years preceding the survey and who had antenatal care visits was included. A multilevel mixed-effects logistic regression model was used to examine the predictors that affect the completion of the continuum of maternity care services. Finally, statistical significance was declared at a *p*-value < 0.05.

**Results:**

In this study, the overall prevalence of completion along the continuum of maternity care was 12.9% (95%CI: 11.1 – 14.9%). Attending higher education (AOR = 2.03: 95%CI; 1.14 - 3.61), belonged to medium wealth status (AOR = 1.69: 95%CI; 1.07 - 2.66), belonged to rich wealth status (AOR = 2.05: 95%CI; 1.32, 3.17), and informed about danger signs during pregnancy (AOR = 2.23: 95%CI; 1.61, 3.10) were positively associated with the completion of the maternity continuum of care. However, late initiaton of first antenatal care visits (AOR = 0.66: 95%CI; 0.49, 0.89), being rural resident (AOR = 0.67: 95%CI; 0.42 - 0.93), lived in the Afar (AOR = 0.36: 95%CI; 0.12 – 0.83) and Gambella (AOR = 0.52: 95%CI; 0.19 – 0.95) regional states were negatively associated with the completion of the continuum of maternity care.

**Conclusion:**

Despite most of the women using at least one of the maternity services, the level of completion along the continuum of care after antenatal care booking remains low in Ethiopia. Therefore, enhancing female education and economic transitions with special consideration given to rural, Afar, and Gambella regional state residents. Counseling towards the danger signs of pregnancy and its complications during antenatal care follow-upshould be strengthened. . Furthermore, the identified predictors should be considered when designing new policies or updating policies and strategies on maternity services uptake to step-up its full utilization, which in turn helps in the achievement of the sustainable development goals of ending preventable causes of maternal, neonatal, and child death by 2030.

## Introduction

Globally, despite several years of focused efforts, maternal and under-five mortality remains a major public health problem [1]. In 2017, about 295,000 maternal death [2], and 2.4 million neonatal deaths in 2019 [3] were recorded, globally. Sub-Saharan Africa (SSA) and Southern Asia shoulder the highest burden of the reported global maternal (86%) and neonatal (80%) mortality [2, 3]. The majority of these maternal and neonatal mortalities occur during childbirth or shortly after delivery [4, 5]. As a result, to improve maternal and neonatal health outcomes, the global development organizations and policymakers recommend the utilization of a continuum of maternity care throughout the antenatal, intrapartum, and postpartum periods [6–10]. According to the World Health Organization (WHO), the "Continuum of Care" for maternity services is defined as a package of integrated services delivered for the mothers and newborns starting from pre-pregnancy to childbirth, the postnatal period, and childhood [11].

The merit of the continuum of care is that each stage builds on the accomplishments of the preceding stage, ensuring a better comprehensive health care experience for women and children [12]. Timely (initiated at the first trimester of pregnancy) [13, 14], frequent (four or more visits) [12, 13, 15], and adequate (with proper contents) [12, 13, 15] antenatal care services provided for the mother during the period of pregnancy reduce the risk of complication and death for the mother as well as the unborn fetus and improve the uptake of subsequent maternal services. Birth attended by skilled professionals reduces maternal as well as neonatal mortality [12, 16]. Postnatal care is very essential to ensuring the women’s and children’s health [12], and it is also a good entry point to initiate postpartum family planning early, which prevents the risks associated with unintended pregnancy and short birth interval [17]. On the other hand, missing from each of the continuum of care is correlated with poor maternal, neonatal, and child health outcomes [18–20].

To improve the uptake of maternal health services in the continuum of care pathway, the government of Ethiopia in collaboration with other non-governmental organizations (NGOs) has implemented different initiatives that expand access to essential health services like primary health care expansion, women development army, health extension program, community-based health insurance, and charge-free maternal health services [21–25]_._ Despite, all these efforts made, the uptake of maternal health care services along the three continuums of care, for instance, antenatal care, institutional delivery, and postnatal care, is one of the lowest as compared to other Sub-Saharan Africa countries [25–27]. As indicated in a secondary data analysis of the 2016 Ethiopia Demographic and Health Survey, only 6.56% of women have completed all the three continuums of maternal care [28] as recommended by WHO [11]. In other fragmented studies conducted in different parts of Ethiopia, the continuum of care ranges from 9.1% [27] to 47% [29]. This portrayed that to improve and strengthen the utilization of maternity services during the continuum of care, factors that became bottlenecks should be identified [30].

Previously studies done in Ethiopia identified that the completion of continuum of maternity care was impending by different factors like age [31], place of residence [31–33], educational status [31–34], marital status [28], religion [28], wealth status [28], parity [28], birth order [28, 33, 35], sex of household head [27], contraceptive use [33], being informed on pregnancy complications/danger signs during pregnancy [28, 31, 34], distance to health facilities [28, 31, 33], knowledge on the continuum of care [33], media exposure [28, 32], woman’s decision making power [31, 33], exposed to health extension program [31], pregnancy-related complication [31], timing of first initiation of ANC [31, 34], and number of ANC visits [31].

However, previously conducted studies in Ethiopia assessed the factors associated with womens’ services uptake in different stages as separate elements either in a design or analysis [36–38], which does not assure that all women receive a package of interventions starting before pregnancy to postpartum period by measuring each maternal service separately [8, 39]. Furthermore, those previous studies were done in specific areas with small sample sizes and were not nationally representative. Moreover, the previous studies primarily focused on individual-level factors with little attention given to community-level factors. However, this could undermine the importance of considering contextual factors when designing appropriate maternal, neonatal, and child health services strategies. Therefore, for bridging all those gaps, this study was aimed to assess the magnitude, and individual and community-level predictors of completion of maternity services along the continuum of care among reproductive-age women using the nationally representative recent Ethiopia mini demographic health survey data. The findings of this study will be indisputable importance to identify the predictors of the complete uptake of maternity services along the continuum of care as a result respective programs are made more appropriately towards the identified predictors and will also have paramount importance to reveal country-levels figures, and screen out modified and persistent predictors, which in turn improve the complete uptake of maternity services along the continuum of care, and achievement of the SDG #3 of ending preventable maternal and neonatal mortality by 2030.

## Methods and materials

### Study setting and period

Secondary data analysis was employed based on the 2019 EmDHS data, which were collected from 21^st^ March/2019 to 28^th^ June/2019. The EDHSs are a nationally representative cross-sectional survey done in nine regions and two city administrations every five years. EmDHS is also a survey conducted in between the standard EDHS (two to three years). Two EmDHSs in 2014 and 2019 have been conducted. For each of the survey, stratified two-stage sampling has been used. In the first stage, stratification was done by region, and then each region was stratified as urban and rural. For 2019 EmDHS data, a total of 305 (94 urban, and 211 rural) enumeration areas (EAs) were selected using probability proportional to EA size in the first stage. In the second stage, households were selected proportionally from each EA by using a systematic sampling method. The detailed method of data collection was accessed at the DHS database [40].

### Data source/extraction

The data were used from the Measure Demographic and Health Surveys (DHS) website (http://www.dhsprogram.com/) after permission was taken through an online request by explaining the objective of the study.

### Population of the study

All women reproductive age women (15 - 49 years) who gave birth in the five years preceding the survey and who had at least one ANC visits for their last child all over Ethiopia were the source population, whereas women who gave birth in the five years preceding the survey and who had at least one ANC visit for their last child and lived in the selected EAs were the study populations.

### Eligibility criteria

All reproductive-age women who gave birth in the five years preceding the survey and found in the selected clusters at least one night before the data collection period were included, whereas, women who gave birth and had no ANC visit were excluded. Finally, a total of 2,905 weighted samples of reproductive-age women were included in this study.

### Variables of the study

#### Dependent variable

The dependent variable was the completion of the continuum. Continuum of maternity care is defined as woman attended at least 4 ANC, had institutional delivery, and PNC services after delivery. If the woman received all mentioned services, she was considered as having complete the continuum of care, labeled as “1”, and if she missed at least one of the recommended services, she was considered as having an incomplete continuum of care, and labeled as “0” [41].

Number of ANC visits was assessed from the question, “How many times you received antenatal care during the last pregnancy?” The information regarding institutional delivery was obtained from the question; “Where did you give birth to the last child?” and women were considered to have institutional delivery if they had a delivery at hospital, health center, clinic or any other health facility. Furthermore, PNC utilization was determined using the questions; “Did anyone check your health after discharge/home delivery?” and/or “In the two months after delivery, did anyone check the health of your child?”. Finally, if the woman responded “yes” for one of the questions, she was considered as she had PNC [42].

#### Independent variables

The independent variables were grouped under individual and community-level variables**.** The individual-level variables include maternal age, marital status, religion, educational status of women, household wealth status, parity, preceding birth interval, sex of household head, number of family size, informed on danger sign, history of poor obstetrics outcome, timing and numbers of antenatal care, place of delivery, mode of delivery, and birth order. Whereas, the place of residence and region of the study participants were classified as community-level variables. The detailed information’s about the explanatory factors were presented in Table 1.

**Table 1:** the description of some of the independent variables

**Variables**	**Definitions/Categories**
**Age of the mothers (in years)**	The age of the mother was coded as 15-19, 20-24, 25-29, 30-34, 35-39, 40-44, and 45-49; however, during data cleaning some of the categories lack or have very limited participants, thus, we regrouped into 15-24, 25-34, and 35-49
**Religion**	Religion was coded as Orthodox, Catholic, Protestant, Muslim, traditional, other. Depending on the number of participants, we sum up Protestant, Catholic, and Traditional, as “Others”. And, we regrouped into Orthodox, Muslim, and others
**Marital status**	Marital status was coded as married, never in the union, living with a partner, widowed, divorced, and no longer living together/separated; but, participants from the last five were very few, so, we categorized in to married and unmarried by living married alone and classified all else.
**Timing of first ANC visit**	The timing of first ANC visit was classified in to “early” if a woman attended ANC visit within the first 12 weeks of gestation, and “late” if she attended ANC visit after 12 weeks of gestation.
**Birth order**	Birth order was a count number ranging from 1 to 15; however, we regrouped into 1^st^, 2^nd^, and third and above
**Parity**	The total number of children ever born was a count number ranging from 1 to 15; however, we regrouped into Primiparous (1), multiparous (2 to 4), and grand-multiparous (≥ 5)
**Preceding birth interval**	Preceding birth interval was coded from 1 to 219 months in the dataset; however, during data cleaning some of the categories lack or have very limited participants, thus, we regrouped into ≤ 23, and ≥ 24 months.
**Number of a household member**	The number of household size was a count number ranging from 1 to 24; however, during data cleaning some of the categories lacked or have very limited participants, so, we recoded into ≤ 5 and ≥ 6.

### Statistical analysis

The data were extracted from the birth record (BR) file data set using SPSS version 24 software and further analyzed using STATA version 15. By using sample weight the data were weighted for probability sampling and non-response to restore the representativeness of the survey and get reliable statistical estimates. Data editing, cleaning, and coding were done. Descriptive statistics were done and presented by tables. Socio-demographic and other profiles of the study participants were compared using the chi-square test. In this study, two levels of data hierarchy were considered due to the sampling technique applied in EDHS (Multistage stratified cluster sampling). Level one unit was the individual pregnant woman in the households and level two units were enumeration areas. Level one (reproductive-age women in the households) was nested within units at the next higher level (enumeration areas). The outcome variable was represented by $${Y}_{ij}=\left\{\begin{array}{c}Completion of the continuum of care\\ Drop out from the continuum of care\end{array}\right.$$, the category is dichotomous. Therefore, the multilevel mixed-effect logistic regression model was fitted to identify the predictors influencing the completion of maternity services along the continuum of care pathway at each level (individual level and community level). For the multilevel logistic regression four models were fitted. The first model (a model without covariate) was fitted to determine the extent of cluster variation in the completion of maternity services along the continuum of the care pathway. The second model was fitted with individual-level factors alone. The third model was fitted with community-level variables. Finally, the fourth model was fitted with both individual and community-level factors. Both bivariable and multivariable analyses were employed. Variables that have a *P*-value of ≤ 0.25 in the bivariable two-level binary logistic regression were candidates for multivariable multilevel logistic regression analysis. Then Variables in multilevel multivariable logistic regression were declared to be statistically significant at a *P*-value of < 0.05. The presence of Multicollinearity among covariates were checked using the variance inflation factor (VIF), and not present. The fitted models were compared based on Akaike’s Information Criteria (AIC) and a model with a small AIC value was nominated and all interpretations and inferences were made based on this model. The random-effects measure the variation of the completion of the continuum of a maternity care pathway across clusters (EAs) and are determined using the Intraclass correlation coefficient (ICC), median odds ratio (MOR), and proportional change in variance (PCV) statistics. The ICC determines the variation within-cluster and between-cluster differences. The PCV determines the total variation of early initiation of ANC at the individual- and community-level factors in each model. The MOR measures the MOR of completion of the continuum of care at the high-risk cluster (clusters having high dropout rate from the continuum of care) and low-risk cluster (clusters having a high prevalence of completion of the continuum of care) when we select randomly two reproductive-age women during data collection from two clusters. The formulas used to tabulate these three measurements are as follows;

$\mathrm{ICC}={\mathrm{V}}_{\mathrm{i}}/\left({\mathrm{V}}_{\mathrm{i}}+{\uppi }^{2} /3\right) \sim \frac{Vi}{Vi+3.29}$, where V_i_ = between cluster (community) variances and π2 /3 = within-cluster (community) variance [43].

$\mathrm{PCV}=\frac{Vi-Vy}{Vi}$, where Vi = variances of the null model, where Vy=variance of the model with more terms [43].

$MOR=exp \left[\sqrt{2\times Vz\times 0.6745}\right]\sim exp.\left[0.95\sqrt{Vz}\right]$ where Vz = variance at the community level [43].

## Results

### Sociodemographic characteristics of the study participants

In this study, a total of 2,905 weighted samples of participants have participated from all over the country. More than half (53.14%) of the study participants were in the age range of 25-34 years old. Of the total study participants, 2,036 (70.08%) were rural residents, 2,717 (93.51%) were married, 1,208 (41.59%) were Orthodox Christian by religion, 1,269 (43.67%) didn’t attend formal education, and 2,519 (86.71%) of the households were headed by males. The majority of the participants were from the Oromia regional state (37%) followed by the Amhara regional state (24.05%) (Table 2).

**Table 2:** Socio-demographic characteristics of reproductive-age women who gave birth in the last five years before the survey in Ethiopia, 2019

**Variable**	**Category**	**Weighted frequency**	**Percentage (%)**
Age (in years)	15-24	752	25.89
	25-34	1,544	53.14
	>=34	609	20.97
Marital	Unmarried	188	6.49
	Married	2,717	93.51
Religion	Orthodox	1,208	41.59
	Muslim	877	30.20
	Others^c^	820	28.22
Region	Addis Ababa	122	4.22
	Tigray	271	9.33
	Afar	32	1.10
	Amhara	698	24.05
	Oromia	1,073	36.95
	Somali	66	2.28
	B/Gumz	39	1.34
	SNNPR	559	19.24
	Gambella	16	0.57
	Harari	9	0.31
	Dire Dawa	17	0.61
Residence	Urban	869	29.92
	Rural	2,036	70.08
Educational status of women	No education	1,269	43.67
	Primary	1,152	39.66
	Secondary	331	11.40
	Higher	153	5.27
Sex of household head	Male	2,519	86.71
	Female	386	13.29
Household wealth status	Poor	977	33.62
	Medium	587	20.21
	Rich	1,341	46.17

Key; ^c^Protestant, catholic or traditional religion follower, *B/Gumz* Benshangul Gumz, *SNNPR* South Nation Nationalities Peoples Region

### Obstetric and reproductive related characteristics of reproductive-age women in Ethiopia

Of the total study participants, more than half (54.62%) of the households have had ≤ 5 family members and nearly 47% were multiparous. The majority (60.15%) of the women had been advised for obstetric danger signs during their last pregnancy. More than sixty percent of the woman have had a birth spacing of 24 months and above, and 62% of the women started their first antenatal care visits lately for the most recent pregnancy (Table 3).

**Table 3:** Obstetric and reproductive related characteristics of reproductive age women in Ethiopia, 2019

**Variable**	**Category**	**Weighted frequency**	**Percentage (%)**
Parity	Primiparous	687	23.64
	Multiparous	1,360	46.83
	Grand multiparous	858	29.53
Giving birth by cesarean section	No	2,297	92.16
	Yes	195	7.84
Timing of first ANC initiation	Early	1,096	37.72
	Late	1,809	62.28
Household members	≤ 5	1,587	54.62
	≥ 6	1,318	45.38
Birth interval (in months)	≤ 23	361	16.48
	≥ 24	1,830	83.52
Age at first birth (in years)	≤17	1,157	39.85
	≥18	1,748	60.15
Danger signs advised during pregnancy	No	1,157	39.85
	Yes	1,748	60.15
Twin	No	2,848	98.03
	Yes	57	1.97
Number of under-five children	No under-five child	97	3.36
	Only one	1,615	55.60
	Two	1,014	34.91
	3 and above	178	6.14
Birth order	First	687	23.64
	Second	613	21.10
	Third and above	1,605	55.26
Number of antenatal care visits	Only one time	130	4.47
	Two times	291	10.02
	Three times	795	27.37
	Four and more	1,689	58.14

### Magnitude of completion of the continuum of care for maternity health services in Ethiopia

**Fig 1:**
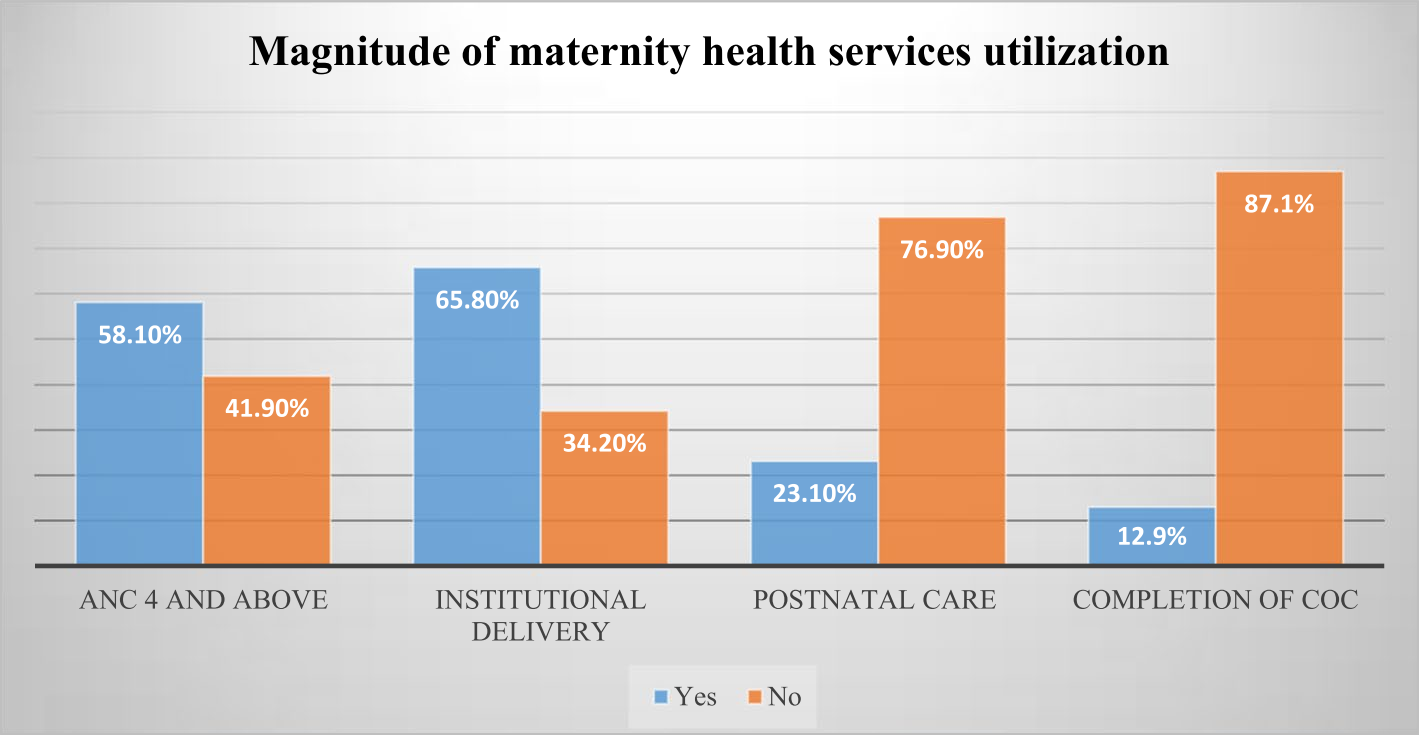
Prevalence of completion of the continuum of maternity care services in Ethiopia, 2019. Key: COC- Continuum of care

In this study, the overall prevalence of completion of the continuum of maternity care was 12.9% (95%CI: 11.1 – 14.9%). Of the total respondents, more than half (58.1%) have had four and above ANC follow-up, and more than two-thirds (65.8%) were delivered at the health institutions. But, only 23.1% of the respondent had postnatal care after discharge/deliver at home within the recommended time period (Fig. 1).

### Completion of the continuum of care by different characteristics of reproductive-age women in Ethiopia

Of the total women who have completed the continuum of care, 47% were in the age group of 25-34 years old, 93% were married, 56.5% were urban residents, 52.2% were multiparous, and 63% of women belonged to rich households. Based on birth interval, the completion of the continuum of care was higher among women who spaced for ≥ 24 months (87.2%). Among women who advised on danger signs during pregnancy, the completion of the continuum of care was 76.4%. Concerning the family size, households having less than six family sizes were experienced a higher prevalence of completion of the continuum of care (57.3%). Regarding the age of first birth, 64.9% of women had completed the continuum of maternity care (Table 4).

**Table 4:** Completion of the continuum of maternity care by different characteristics of reproductive age women in Ethiopia, 2019

**Variable**	**Category**	**Completion of the continuum of maternity care**	
		**No (%)**	**Yes (%)**
Age (in years)	15-24	639 (25.28)	112. (29.92)
	25-34	1367 (54.05)	176 (47)
	>=34	521 (20.7)	86 (23)
Marital	Unmarried	163 (6.5)	25 (6.6)
	Married	2365 (93.5)	349 (93.4)
Religion	Orthodox	1033 (40.8)	175 (46.6)
	Muslim	748 (29.6)	128 (34.2)
	Others^c^	747 (29.5)	72 (19.2)
Region	Addis Ababa	92 (3.7)	30 (7.9)
	Tigray	227 (9.0)	44 (11.7)
	Afar	31 (1.2)	1 (0.4)
	Amhara	615 (24.3)	83 (22.1)
	Oromia	939 (37.1)	134 (35.8)
	Somali	60 (2.4)	6 (1.5)
	B/Gumz	30 (1.2)	8 (2.2)
	SNNPR	494 (19.6)	63 (16.8)
	Gambella	15 (0.6)	1 (0.3)
	Harari	8 (0.3)	1 (0.3)
	Dire Dawa	14 (0.6)	3 (0.9)
Residence	Urban	705 (28)	163 (43.5)
	Rural	1823 (72)	212 (56.5)
Educational status of women	No education	1137 (45)	131 (35)
	Primary	1018 (40)	133 (35.5)
	Secondary	257 (10)	74 (19.7)
	Higher	116 (5)	36 (9.7)
Sex of household head	Male	2194 (87)	323 (86.2)
	Female	334 (13)	52 (13.8)
Last birth by cesarean section	No	2035 (93.8)	261 (81.1)
	Yes	134 (6.2)	61 (18.9)
Timing of first ANC initiation	Early	916 (36.2)	179 (47.7)
	Late	1612 (63.8)	196 (52.3)
Household members	≤ 5	1371 (54.2)	215 (57.3)
	≥ 6	1158 (45.8)	160 (42.7)
Birth interval (in months)	≤ 23	326 (17)	34 (12.8)
	≥ 24	1597 (83)	233 (87.2)
Parity	Primiparous	584 (23.1)	101 (27)
	Multiparous	1164 (46)	196 (52.2)
	Grand multiparous	780 (30.8)	78 (20.7)
Household wealth status	Poor	902 (35.7)	74 (19.7)
	Medium	523 (20.7)	64 (17.1)
	Rich	1103 (43.6)	237 (63.2)
Age at First Birth	≤17	1025 (40.6)	132 (35.1)
	≥18	1503 (59.4)	243 (64.9)
Advised about danger signs during pregnancy	No	1069 (42.3)	88 (23.6)
	Yes	1459 (57.7)	287 (76.4)
Twin	No	2479 (98)	368 (98)
	Yes	49 (2)	8 (2)
Number of under-five children	No	80 (3.2)	16 (4.3)
	Only one	1389 (54.9)	225 (60.1)
	Two	890 (35.2)	123 (32.9)
	3 and above	168 (6.6)	10 (2.7)

Key; ^c^Protestant, catholic or traditional religion follower, *B/Gumiz* Benshangul-Gumz region, *SNNPR* South Nation Nationality peoples region

### Random effect and model comparison

The Intraclass correlation coefficient (ICC) in the null model was (0.197), which means that 19.7% of the variability of completion of the continuum of maternity care was due to the differences between clusters or unobserved factors at the community level. This noted that the multilevel logistic regression model is better to estimate the completion of a continuum of care among reproductive-age women who gave birth in the last five years and had booked ANC visits than single-level logistic regression. The Akaike’s Information Criteria (AIC) is smallest at model 4 (AIC = 1532.4) as compared to random intercept only model or null model (AIC = 2217.2), a model with only individual-level factors (AIC = 1536.2), and model with only community-level factors (AIC = 2174.6). Therefore this model is the best-fitted model for the data because it has the smallest AIC as compared to the rest models. As a result, all interpretations and reports were made based on this model. In addition, the median odds ratio (MOR) in all models was greater than one noted that there is a variation in completion of the continuum of care between community levels (clusters). The value of MOR (2.31) in the null model depicts that there was a variation of completion of the continuum of care between clusters when we randomly select women from two clusters, women from clusters that have a high prevalence of completion of the continuum of care were 2.31 times more likely to complete continuum of care as compared to women from low prevalence of continuum of care cluster. The higher proportional change in variance (PCV) value in the fourth model (47.5%) depicted that about 47.5% of the variability of completion of the continuum of care was explained by both the individual-level and community-level factors (Table 5).

**Table 5:** Random effect and model comparison for predictors of completion of maternity care among reproductive-age women in Ethiopia, 2019

**Parameter**	**Null (model I)**	**Model II**	**Model III**	**Model IV**
ICC	19.7%	15.3%	12.7%	11.5%
Variance	0 .80 (0.53, 1.23)	0.59 (0.32, 1.08)	0.48 (0.28, 0.82)	0.42 (0.19, 0.91)
MOR	2.31	1.98	1.79	1.67
PCV	Reference	26%	40%	47.5%
Model fitness				
AIC	2217.2	1536.2	2174.6	1532.4

*AIC* Akaike’s information criteria, *ICC* Intraclass correlation coefficient, *MOR* Median Odds ratio, *PCV* Proportional change in variance

### Predictors of the completion of the continuum of maternity care in Ethiopia

As noted in the final model (model 4) both individual and community-level factors were added for multilevel analysis, of which, maternal educational status, household wealth status, the timing of the first initiation of antenatal care visit, danger sign advised during pregnancy, residence, and region were significantly associated with completion of maternity care uptake in Ethiopia at *P-*value < 0.05 (Table 4). The odds of completion of the continuum of care for the maternity services were two times (AOR = 2.03: 95%CI; 1.14 - 3.61) more likely among women who attended higher educations as compared to those women who didn’t attend formal education. Women who belonged to the household wealth status of medium, and rich were 1.7 (AOR = 1.69: 95%CI; 1.07 - 2.66), and 2 (AOR = 2.05: 95%CI; 1.32, 3.17) times more likely to complete the continuum of maternity care as compared to women who belonged to poor wealth status, respectively. Women who had advised on danger signs of pregnancy during their ANC visits were 2 (AOR = 2.23: 95%CI; 1.61, 3.10) times more likely to complete the continuum of maternity care than their counterparts. Whereas, the odds of the completion of the continuum of maternity care was 34% (AOR = 0.66: 95%CI; 0.49, 0.89) less likely among women who attended their first ANC visits late as compared to women who attended their first ANC visits early. Likewise, the odds of completion of the continuum of maternity care was 33% (AOR = 0.67: 95%CI; 0.42 - 0.93) less likely among women who resided in rural areas as compared to their counterparts. Women who lived in the Afar and Gambella regional states were 64% (AOR = 0.36: 95%CI; 0.12 – 0.83), and 48% (AOR = 0.52: 95%CI; 0.19 – 0.95) less likely to complete the continuum of maternity care than women who lived in Addis Ababa, the capital city of Ethiopia (Table 6).

**Table 6:** Multilevel mixed-effect logistic regression analysis to assess the predictors of the continuum of maternity care among women in Ethiopia, 2019

**Variable**	**Category**	**Null (model I)**	**Model II** AOR (95%CI)	**Model III** AOR (95%CI)	**Model VI** AOR (95%CI)
Maternal age (in years)	15-24	-	1	-	1
	25-34	-	1.01 (0.62, 1.64)	-	0.96 (0.59, 1.56)
	35-49	-	1.17 (0.65, 2.1)	-	1.07 (0.59, 1.93)
Marital status	Unmarried	-	1	-	1
	Married	-	1.31 (0.64, 2.64)	-	1.34 (.66, 2.73)
Religion	Orthodox	-	1	-	1
	Muslim	-	1.03 (0.72, 1.48)	-	1.15 (0.75, 1.77)
	Others^c^	-	0.63 (0.39, 1.01)	-	0.73 (0. 42, 1.29)
Educational status	No education	-	1	-	1
	Primary	-	1.27 (0.90, 1.79)	-	1.22 (0.86, 1.73)
	Secondary	-	1.21 (0.71, 2.07)	-	1.16 (0.68, 2.00)
	Higher	-	2.27 (1.29, 3.98)	-	2.03 (1.14, 3.61)*
Household wealth status	Poor	-	1	-	1
	Medium	-	1.89 (1.20, 2.98)	-	1.69 (1.07, 2.66)*
	Rich	-	2.31 (1.55, 3.44)	-	2.05 (1.32, 3.17)*
Sex of household head	Male	-	1	-	1
	Female	-	1.02 (0.67, 1.54)	-	1.12 (0.74, 1.71)
Birth interval (in months)	< 24	-	1	-	1
	≥ 24	-	1.30 (0.86, 1.96)	-	1.25 (0.83, 1.88)
Family size	≤ 5	-	1	-	1
	≥ 6	-	1.01 (0.73, 1.39)	-	1.01 (0.74, 1.40)
Birth order	1^st^ or Second	-	1	-	1
	Third or above	-	1.19 (0.80, 1.75)	-	1.21 (0.82, 1.78)
Age at first birth (in years)	< 18	-	1	-	1
	≥ 18	-	0.94 (0.68, 1.29)	-	0.92 (0.67, 1.27)
Initiation of first antenatal care (in months)	≤ 3	-	1	-	1
	>3	-	0.65 (0.49, 0.88)	-	0.66 (0.49, 0.89)*
During pregnancy danger signs advised	No	-	1	-	1
	Yes	-	2.31 (1.67, 3.19)	-	2.23 (1.61, 3.10)*
Twin	No	-	1	-	1
	Yes	-	1.05 (0.37, 3.00)	-	1.04 (0.37, 2.93)
Community-level variables					
Residence \	Urban	-	-	1	1
	Rural	-	-	0.46(0.32,0 .66)	0.67 (0.42, 0.93)*
Region	Addis Ababa	-	-	1	1
	Tigray	-	-	0.92 (0.47, 1.80)	1.06 (0.48, 2.36)
	Afar	-	-	0.21 (0.08, 0.51)	0.36 (0.12, 0.83)*
	Amhara	-	-	0.82 (0.42, 1.61)	1.36 (0.62, 2.99)
	Oromia		-	0.74 (0.38, 1.44)	0 .92 (0.41, 2.09)
	Somalia		-	0.37 (0.13, 1.02)	0.54 (0.14, 1.96)
	B/Gumz		-	1.49 (0.76, 2.94)	2.30 (0.91, 5.05)
	SNNPR		-	0.68 (0.34, 1.36)	1.19 (0.52, 2.78)
	Gambella		-	0.34 (0.16, 0.75)	0.52 (0.19, 0.95)*
	Harari		-	0.73 (0.38, 1.39)	0.77 (0.35, 1.65)
	Dire Dawa		-	0.93 (0.49, 1.78)	1.16 (0.53, 2.51)

^*^p-value < 0.05, *AOR* Adjusted odds ratio, *CI* Confidence interval, *1*: Reference, ^c^Protestant, catholic or traditional religion follower, *SNNPR* South Nation Nationality peoples region, *B/Gumiz* Benshangul-Gumz region

## Discussion

The continuum of care along maternity services is recognized as one of the pillar packages to enhance good maternal and neonatal health outcomes and to achieve the SDG goal which indicated under ending preventable causes of maternal, neonatal, and child death by 2030 [1, 29]. Therefore, this study gives information about the magnitude and predictors of completion of the continuum of maternity care in Ethiopia using the 2019 intermediate Ethiopia mini demographic and health survey.

The study found that the overall magnitude of the completion of the continuum of maternity care was 12.9% (95%CI: 11.1 – 14.9%). This result was in line with studies done in Ethiopia (13.9%) [31], Nigeria (11.7%) [44], and Sub-Saharan Africa (13.6%) [45]. However, the finding was higher than studies done in Ethiopia, for instance, Arbaminch (9.7%) [46] and EDHS 2016 (6.56%) [28]. It was also higher than studies done in Ghana (8%) [47], and Tanzania (10%) [48]. This variation could be due to the difference in the gaps in the study period [28, 47, 48], coverage of only rural residents [46], the launching and strengthened functioning of the Health Extension Program (HEP) and women development army, and improving access to health care systems in the country.

On the other hand, the current study finding was lower than studies conducted in Ethiopia [29, 33, 34], Nepal (45.7%) [49], Cambodia (60%) [35], Egypt (50.4) [50] , and Pakistan (27%) [51]. The discrepancies could be due to the difference in the study settings such that sample size, study area [35, 49–51]^,^ and study populations; in which most of the participants in the previous studies [29, 34] were urban dwellers whereas more than 70% of the mothers who participated in the current study were rural residents. So, the lower prevalence of completion of the continuum of maternity care reported in this study might be explained by women who lived in rural areas are less likely to have a nearby health facility which in turn exposed to other extra costs for transportation service as well as lack of availability of means of transportations. As a reason, they fail to attain the completion of the continuum of maternity care. In addition, the other possible explanation might be the difference in the operational definition used to classify the outcome variable of the continuum of care [29].

Completion of the continuum of maternity care was positively influenced by women’s educational status. The odds of completion along the continuum of maternity care among women who attended higher educations were more likely than those women who didn’t attend formal education. This finding was in agreement with studies done in Ethiopia [32, 34, 52], Egypt [50], Nepal [49], and Pakistan [51]. This could be justified by the fact that education [34, 53, 54] has been proven to change the traditional balance of power within the household, leveraging change in the decision-making power and distribution of resources within the family, modifying their level of understanding about accruing and treating disease, influencing on the use of modern health care practices, economic transitions, and autonomy. As a result, the women who has being educated were more likely to complete the continuum of maternity care than a woman who didn’t attend formal education.

Consistent with other studies [28, 35, 51], women who belonged to the household wealth status of medium, and rich were more likely to complete the continuum of maternity care as compared to women who belonged to poor wealth status. Though maternity services (i.e. antenatal care, skilled birth attendants, and postnatal care) are provided free of charge for all women in Ethiopia at government health facilities, services fees at private health facilities, and non-services related costs like transportation fees [55, 56] are high. In addition, most women are obliged to experience a long waiting time to get the services, and going a long distance to and from health facilities. Such types of indirect costs are linked with the women’s daily life of which they might go to a farm, market, office, and other workplaces to gain money to cover their daily living. As a result, women belonging to middle and rich households will be more likely to complete the continuum of maternity care as compared to women belonging to the poor wealth index.

Consistent with other studies [27, 34], women who have been informed about danger signs during pregnancy by the time of their ANC visits were more likely to complete the continuum of care for maternity health services than their counterparts. This might be because women who have been advised on danger signs during pregnancy are a higher likelihood of recognizing the bad consequences of it and the benefit of giving birth at equipped health facilities and timely uptake of maternal and neonatal health services for any complications [57].

Whereas, the odds of the completion of the continuum of maternity care were 34% less likely among women who started their first ANC visits late as compared to those women who started their first ANC visits early. This finding was supported with other study findings at which women who initiated their first ANC visits early were more likely to complete the continuum of care than their counterparts [29, 34]. The possible justification for this might be women who initiate first ANC visits at late gestational age have a lower likelihood of knowing a correct gestational age and the expected date of delivery, the poor likelihood of having a birth plan regarding the place of birth, preferred birth attendants, birth companion, means of transport and blood donor if needed [34, 58, 59]. On the other hand, respondents who start antenatal care in the early gestational age have a higher chance to contact the health care provider and have been exposed to information which increases the women's level of knowledge towards a place of delivery and having post-partum care [29].

Completion of the continuum of maternity care was negatively correlated with the rural residence. Similar to other studies [29, 45, 47, 60, 61], the odds of completion of the continuum of maternity care were less likely among women who resided in rural areas than women who lived in urban. This could be due to women who resided in rural areas are experiencing inadequate availability and accessibility of health facilities, and fewer chances of getting health information as compared to women who lived in urban areas. Moreover, women who resided in the urban area are more likely to be educated [62]. As a reason, women who resided in rural areas were less likely to complete the continuum of maternity care than women who resided in urban areas.

There were regional variations among completion of the continuum of maternity care. Women who lived in the Afar and Gambella regional states were less likely to complete the continuum of maternity care as compared with women who lived in Addis Ababa, the capital city of the country. The variation could be due to the difference in accessibility and availability of health facilities; in which participants from Addis Ababa are more socioeconomically developed, and have better accessibility, and availability of health facilities, including both public and private health services [63].

The clinical and public health implication of this study is to maximize the continuum of maternity care and to decrease the feto-maternal mortality and morbidity that occurred due to missed opportunities. As a result, giving special attention to women from rural areas, women with no formal education, women from poorer household wealth status, and Afar and Gambella regional states could maximize the completion of the continuum of maternity care in reproductive-age women.

### Strength and limitation of the study

The study had many strengths, for instance, it used nationalily representative data of the Ethiopia demographic health survey with a large sample size, a high response rate, and high-quality data which could minimize sampling and measurement-related bias. Moreover, we have used an appropriate statistical approach, multilevel mixed-effect analysis, to estimate the cluster effect on completion of the continuum of maternity care. As a limitation, owing to the cross-sectional nature of the study, the exact cause-effect relationship between completion of the continuum of maternity care and its predictors doesn’t exist and recall bias might be introduced. The other limitation was the study failed to assess some important variables like distance to the health facility, pregnancy intention, and media exposure which may affect the completion of the continuum of care. The study also failed to assess the effect of utilization of one basic maternal health service on the next MCH service.

## Conclusion

In conclusion, the study demonstrated a lower rate of completion along the maternity continuum of care in Ethiopia. The study also revealed that education, household wealth status, and advice about danger signs during pregnancy were a strong positive association with completion of the continuum of maternity care. However, women who started first ANC visits late, being rural residents, and living in the Afar and Gambella regional states were negatively associated with the completion of the continuum of maternity care. Therefore, enhancing female education and economic transitions with special consideration given to rural, Afar, and Gambella regional state residents could maximize the complete uptake of the recommended maternity services. Counseling towards the danger signs of pregnancy and its complications during antenatal care follow-up should be provided much strengthened to maximize full utilization of the continuum of care. Furthermore, to increase the complete uptake of maternity care services the identified predictors should be considered when designing new policies or updating policies and strategies on maternity services uptake to step-up its full utilization, which in turn help in the reduction of maternal and neonatal mortality and to achieve the aim of the sustainable development goals 3 by 2030.

## Data Availability

The data were accessed from the Measure DHS website (http://www.dhsprogram.com) after permission was obtained through an online request by explaining the objective of this study. The datasets analyzed during this study are available from the corresponding author upon reasonable request.

## Abbreviations

ANC: Antenatal Care
AOR: Adjusted odds ratio
CI: Confidence Interval
SNNPR: South Nation Nationality Peoples Region
WHO: World Health Organization

## References

[1] United Nations. Transforming our World: The 2030 Agenda for Sustainable Development. 2015. Available at: https://sdgs.un.org/2030agenda.

[2] World Health Organization. Trends in maternal mortality 2000 to 2017: estimates by WHO, UNICEF, UNFPA, World Bank Group and the United Nations Population Division: executive summary. World Health Organization. 2019. https://apps.who.int/iris/handle/10665/327596. License: CC BY-NC-SA 3.0 IGO.

[3] UNICEF: United Nations Inter-agency Group for Child Mortality Estimation (UN IGME) 2020. Available at: https://data.unicef.org/topic/child-survival/under-five-mortality/ Released at Augest/2021.

[4] Kassebaum NJ, Barber RM, Bhutta ZA, Dandona L, Gething PW, Hay SI, Kinfu Y, Larson HJ, Liang X, Lim SS (2016). Global, regional, and national levels of maternal mortality, 1990–2015: a systematic analysis for the Global Burden of Disease Study 2015. Lancet.

[5] WHO: Neonatal mortality [Internet]. Geneva, Switzerland: World Health Organization; 2016. Available at: http://www.who.int/gho/child_health/mortality/neonatal_text/en/.

[6] Campbell OM, Graham WJ, group LMSSs (2006). Strategies for reducing maternal mortality: getting on with what works. The lancet.

[7] Filippi V, Ronsmans C, Campbell OM, Graham WJ, Mills A, Borghi J, Koblinsky M, Osrin D (2006). Maternal health in poor countries: the broader context and a call for action. The Lancet.

[8] Kerber KJ, de Graft-Johnson JE, Bhutta ZA, Okong P, Starrs A, Lawn JE (2007). Continuum of care for maternal, newborn, and child health: from slogan to service delivery. The Lancet.

[9] UNICEF, and World Health Organization. A decade of tracking progress for maternal, newborn and child survival: the 2015 report. Countdown to 2015. 2015. Available at: https://www.countdown2030.org/2015/2015-final-report.

[10] World Health Organization. WHO recommendations on maternal health: guidelines approved by the WHO Guidelines Review Committee. 2017. Available at: https://www.who.int/publications/i/item/WHO-MCA-17.10. 40455892

[11] WHO: PMNCH Fact Sheet: RMNCH Continuum of care. Available at: https://www.who.int/pmnch/about/continuum_of_care/en/. 2011.

[12] Marchant T, Tilley-Gyado RD, Tessema T, Singh K, Gautham M, Umar N, Berhanu D, Cousens S, Armstrong Schellenberg JR (2015). Adding content to contacts: measurement of high quality contacts for maternal and newborn health in Ethiopia, north east Nigeria, and Uttar Pradesh, India. PLoS One.

[13] Heredia-Pi I, Servan-Mori E, Darney BG, Reyes-Morales H, Lozano R (2016). Measuring the adequacy of antenatal health care: a national cross-sectional study in Mexico. Bull World Health Organ.

[14] Sinyange N, Sitali L, Jacobs C, Musonda P, Michelo C (2016). Factors associated with late antenatal care booking: population based observations from the 2007 Zambia demographic and health survey. Pan Afr Med J.

[15] Fekadu GA, Ambaw F, Kidanie SA (2019). Facility delivery and postnatal care services use among mothers who attended four or more antenatal care visits in Ethiopia: further analysis of the 2016 demographic and health survey. BMC Pregnancy Childbirth.

[16] Moyer CA, Mustafa A (2013). Drivers and deterrents of facility delivery in sub-Saharan Africa: a systematic review. Reprod Health.

[17] Baqui AH, Ahmed S, Begum N, Khanam R, Mohan D, Harrison M, Al Kabir A, McKaig C, Brandes N, Norton M (2018). Impact of integrating a postpartum family planning program into a community-based maternal and newborn health program on birth spacing and preterm birth in rural Bangladesh. J Global Health.

[18] Lassi ZS, Majeed A, Rashid S, Yakoob MY, Bhutta ZA (2013). The interconnections between maternal and newborn health–evidence and implications for policy. J Matern Fetal Neonatal Med.

[19] Bashour HN, Kharouf MH, AbdulSalam AA, El Asmar K, Tabbaa MA, Cheikha SA (2008). Effect of postnatal home visits on maternal/infant outcomes in Syria: a randomized controlled trial. Public Health Nurs.

[20] Ezeh OK, Agho KE, Dibley MJ, Hall J, Page AN (2014). Determinants of neonatal mortality in Nigeria: evidence from the 2008 demographic and health survey. BMC Public Health.

[21] Lindtjørn B, Mitiku D, Zidda Z, Yaya Y (2017). Reducing maternal deaths in Ethiopia: results of an intervention Programme in Southwest Ethiopia. PLoS One.

[22] Pearson L, Gandhi M, Admasu K, Keyes EB (2011). User fees and maternity services in Ethiopia. Int J Gynecol Obstet.

[23] USAID: USAID invests in improving the quality of maternal and child health services across the country. Available at: https://www.usaid.gov/ethiopia/global-health/maternal-and-child-health. Released on 12 Jul 2021.

[24] Medhanyie A, Spigt M, Kifle Y, Schaay N, Sanders D, Blanco R, GeertJan D, Berhane Y (2012). The role of health extension workers in improving utilization of maternal health services in rural areas in Ethiopia: a cross sectional study. BMC Health Serv Res.

[25] Wang H, Tesfaye R, Ramana GNV, Tesfaye CC. Ethiopia Health Extension Program: An Institutionalized Community Approach for Universal Health Coverage. World Bank Studies; Washington, DC: World Bank. © World Bank; 2016. https://openknowledge.worldbank.org/handle/10986/24119. License: CC BY 3.0 IGO.

[26] Federal Minister of Health (FMOH), National strategy for newborn and child survival in Ethiopia (2015/16–2019/20), Addis Ababa, Ethiopia; 2015. Available at: https://www.healthynewbornnetwork.org/resource/national-strategy-newborn-child-survival-ethiopia/.

[27] Chaka EE, Parsaeian M, Majdzadeh R (2019). Factors associated with the completion of the continuum of care for maternal, newborn, and child health services in Ethiopia. Multilevel model analysis. Int J Prev Med.

[28] Muluneh AG, Kassa GM, Alemayehu GA, Merid MW (2020). High dropout rate from maternity continuum of care after antenatal care booking and its associated factors among reproductive age women in Ethiopia, Evidence from Demographic and Health Survey 2016. PLoS One.

[29] Asratie MH, Muche AA, Geremew AB (2020). Completion of maternity continuum of care among women in the post-partum period: Magnitude and associated factors in the northwest, Ethiopia. PLoS One.

[30] Alkema L, Chou D, Hogan D, Zhang S, Moller A-B, Gemmill A, Fat DM, Boerma T, Temmerman M, Mathers C (2016). Global, regional, and national levels and trends in maternal mortality between 1990 and 2015, with scenario-based projections to 2030: a systematic analysis by the UN Maternal Mortality Estimation Inter-Agency Group. Lancet.

[31] Dadi TL, Medhin G, Kasaye HK, Kassie GM, Jebena MG, Gobezie WA, Alemayehu YK, Teklu AM (2021). Continuum of maternity care among rural women in Ethiopia: does place and frequency of antenatal care visit matter?. Reprod Health.

[32] Tsega D, Admas M, Talie A, Tsega TB, Birhanu MY, Alemu S, Mengist B (2022). Maternity Continuum Care Completion and Its Associated Factors in Northwest Ethiopia. J Pregnancy.

[33] Sertsewold SG, Debie A, Geberu DM (2021). Continuum of maternal healthcare services utilisation and associated factors among women who gave birth in Siyadebirena Wayu district, Ethiopia: community-based cross-sectional study. BMJ Open.

[34] Tizazu MA, Sharew NT, Mamo T, Zeru AB, Asefa EY, Amare NS (2021). Completing the Continuum of Maternity Care and Associated Factors in Debre Berhan Town, Amhara, Ethiopia, 2020. J Multidiscip Healthc.

[35] Wang W, Hong R (2015). Levels and determinants of continuum of care for maternal and newborn health in Cambodia-evidence from a population-based survey. BMC Pregnancy Childbirth.

[36] Tesfaye G, Loxton D, Chojenta C, Semahegn A, Smith R (2017). Delayed initiation of antenatal care and associated factors in Ethiopia: a systematic review and meta-analysis. Reprod Health.

[37] Alemi Kebede KH, Teklehaymanot AN (2016). Factors associated with institutional delivery service utilization in Ethiopia. Int J Women's Health.

[38] Chaka EE, Abdurahman AA, Nedjat S, Majdzadeh R (2019). Utilization and determinants of postnatal care services in Ethiopia: a systematic review and meta-analysis. Ethiopian J Health Sci.

[39] Bryce J, Arnold F, Blanc A, Hancioglu A, Newby H, Requejo J, Wardlaw T, Measurement CWGoIC (2013). Measuring coverage in MNCH: new findings, new strategies, and recommendations for action. PLoS Med.

[40] Institute EPH, ICF (2019). Ethiopia mini demographic and health survey 2019: key indicators.

[41] Emiru AA, Alene GD, Debelew GT (2020). Women’s retention on the continuum of maternal care pathway in west Gojjam zone, Ethiopia: multilevel analysis. BMC Pregnancy Childbirth.

[42] Ayele BG, Woldu MA, Gebrehiwot HW, Gebre-Egziabher EG, Gebretnsae H, Hadgu T, Abrha AA, Medhanyie AA (2019). Magnitude and determinants for place of postnatal care utilization among mothers who delivered at home in Ethiopia: a multinomial analysis from the 2016 Ethiopian demographic health survey. Reprod Health.

[43] Merlo J, Chaix B, Yang M, Lynch J, Råstam L (2005). A brief conceptual tutorial of multilevel analysis in social epidemiology: linking the statistical concept of clustering to the idea of contextual phenomenon. J Epidemiol Community Health.

[44] Akinyemi JO, Afolabi RF, Awolude OA (2016). Patterns and determinants of dropout from maternity care continuum in Nigeria. BMC Pregnancy Childbirth.

[45] Singh K, Story WT, Moran AC (2016). Assessing the continuum of care pathway for maternal health in South Asia and sub-Saharan Africa. Matern Child Health J.

[46] Haile D, Kondale M, Andarge E, Tunje A, Fikadu T, Boti N (2020). Level of completion along continuum of care for maternal and newborn health services and factors associated with it among women in Arba Minch Zuria woreda, Gamo zone, Southern Ethiopia: a community based cross-sectional study. PLoS One.

[47] Yeji F, Shibanuma A, Oduro A, Debpuur C, Kikuchi K, Owusu-Agei S, Gyapong M, Okawa S, Ansah E, Asare GQ (2015). Continuum of care in a maternal, newborn and child health program in Ghana: low completion rate and multiple obstacle factors. PLoS One.

[48] Mohan D, LeFevre AE, George A, Mpembeni R, Bazant E, Rusibamayila N, Killewo J, Winch PJ, Baqui AH (2017). Analysis of dropout across the continuum of maternal health care in Tanzania: findings from a cross-sectional household survey. Health Policy Plan.

[49] Tamang TM: Factors associated with completion of continuum of Care for Maternal Health in Nepal. In: IUSSP XXVIII International Population Conference, Cape Town, South Africa: 2017; 2017.

[50] Hamed A, Mohamed E, Sabry M (2018). Egyptian status of continuum of care for maternal, newborn, and child health: Sohag Governorate as an example. Int J Med Sci Public Health.

[51] Iqbal S, Maqsood S, Zakar R, Zakar MZ, Fischer F (2017). Continuum of care in maternal, newborn and child health in Pakistan: analysis of trends and determinants from 2006 to 2012. BMC Health Serv Res.

[52] Shitie A, Assefa N, Dhressa M, Dilnessa T (2020). Completion and factors associated with maternity continuum of care among mothers who gave birth in the last one year in Enemay district, Northwest Ethiopia. J Pregnancy.

[53] Weitzman A (2017). The effects of women's education on maternal health: Evidence from Peru. Soc Sci Med.

[54] Elo IT (1992). Utilization of maternal health-care services in Peru: the role of women's education. Health Trans Rev.

[55] Gong E, Dula J, Alberto C, de Albuquerque A, Steenland M, Fernandes Q, Cuco RM, Sequeira S, Chicumbe S, Gudo ES (2019). Client experiences with antenatal care waiting times in southern Mozambique. BMC Health Serv Res.

[56] Kalu-Umeh NN, Sambo MN, Idris SH, Kurfi AM (2013). Costs and patterns of financing maternal health care services in rural communities in northern Nigeria: evidence for designing national fee exemption policy. Int J MCH AIDS.

[57] World Health Organization. WHO recommendations on antenatal care for a positive pregnancy experience: World Health Organization. 2016. Available at: https://www.who.int/publications/i/item/9789241549912. 28079998

[58] Atisa FO. Influence of early booking for antenatal care on antenatal and early pregnancy outcomes at Kenyatta National Hospital. University of Nairobi. 2015. Available at: https://www.ajol.info/index.php/jogeca/article/view/150892.

[59] Tiruneh GT, Worku A, Berhane Y, Betemariam W, Demissie M (2020). Determinants of postnatal care utilization in Ethiopia: a multilevel analysis. BMC Pregnancy Childbirth.

[60] Wang W, Hong R (2013). The continuum of care for maternal and newborn health in Cambodia: where are the gaps and why? A population-based study. Lancet.

[61] Dadi LS, Berhane M, Ahmed Y, Gudina EK, Berhanu T, Kim KH, Getnet M, Abera M (2019). Maternal and newborn health services utilization in Jimma Zone, Southwest Ethiopia: a community based cross-sectional study. BMC Pregnancy Childbirth.

[62] World Health Organization. World health statistics 2016: monitoring health for the SDGs, sustainable development goals. World Health Organization; 2016. https://apps.who.int/iris/handle/10665/206498.

[63] Yeneneh A, Alemu K, Dadi AF, Alamirrew A (2018). Spatial distribution of antenatal care utilization and associated factors in Ethiopia: evidence from Ethiopian demographic health surveys. BMC Pregnancy Childbirth.

